# Free Light Chains *κ* and *λ* as New Biomarkers of Selected Diseases

**DOI:** 10.3390/ijms24119531

**Published:** 2023-05-31

**Authors:** Monika Gudowska-Sawczuk, Barbara Mroczko

**Affiliations:** 1 Department of Biochemical Diagnostics, Medical University of Bialystok, Waszyngtona 15A St., 15-269 Bialystok, Poland; 2 Department of Neurodegeneration Diagnostics, Medical University of Bialystok, Waszyngtona 15A St., 15-269 Bialystok, Poland

**Keywords:** free light chains, *κ*, *λ*, inflammation, biomarker, multiple sclerosis, cancer, viral infections, diabetes, cardiovascular disorders, rheumatic disease

## Abstract

Diagnostic and prognostic markers are necessary to help in patient diagnosis and the prediction of future clinical events or disease progression. As promising biomarkers of selected diseases, the free light chains (FLCs) *κ* and *λ* were considered. Measurements of FLCs are currently used in routine diagnostics of, for example, multiple myeloma, and the usefulness of FLCs as biomarkers of monoclonal gammopathies is well understood. Therefore, this review focuses on the studies concerning FLCs as new potential biomarkers of other disorders in which an inflammatory background has been observed. We performed a bibliometric review of studies indexed in MEDLINE to assess the clinical significance of FLCs. Altered levels of FLCs were observed both in diseases strongly connected with inflammation such as viral infections, tick-borne diseases or rheumatic disorders, and disorders that are moderately associated with immune system reactions, e.g., multiple sclerosis, diabetes, cardiovascular disorders and cancers. Increased concentrations of FLCs appear to be a useful prognostic marker in patients with multiple sclerosis or tick-borne encephalitis. Intensive synthesis of FLCs may also reflect the production of specific antibodies against pathogens such as SARS-CoV-2. Moreover, abnormal FLC concentrations might predict the development of diabetic kidney disease in patients with type 2 diabetes. Markedly elevated levels are also associated with increased risk of hospitalization and death in patients with cardiovascular disorders. Additionally, FLCs have been found to be increased in rheumatic diseases and have been related to disease activity. Furthermore, it has been suggested that inhibition of FLCs would reduce the progression of tumorigenesis in breast cancer or colitis-associated colon carcinogenesis. In conclusion, abnormal levels of *κ* and *λ* FLCs, as well as the ratio of *κ*:*λ*, are usually the result of disturbances in the synthesis of immunoglobulins as an effect of overactive inflammatory reactions. Therefore, it seems that *κ* and *λ* FLCs may be significant diagnostic and prognostic biomarkers of selected diseases. Moreover, the inhibition of FLCs appears to be a promising therapeutical target for the treatment of various disorders where inflammation plays an important role in the development or progression of the disease.

## 1. Introduction

Immunoglobulins are the most important proteins of the specific immune response that are produced by plasma cells. The task of antibodies is to protect the body from the negative effects of various damaging factors, such as microorganisms. Immunoglobulins are found in the body fluids of all vertebrates and are produced upon contact with antigens, or in some cases, even after contact with the body’s own tissues (autoantigens).

**Figure 1:**
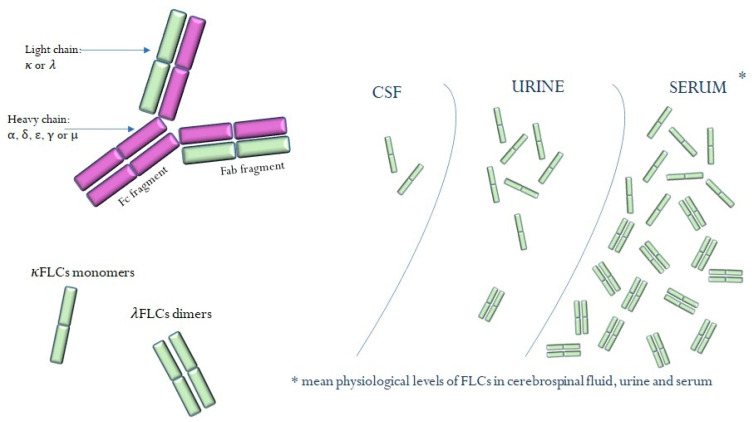
Free light chains.

Each immunoglobulin is Y-shaped and is made up of four polypeptide chains: two light and two heavy. One light chain linked to a part of a heavy chain creates the Fab fragment which contains the paratope (antigen-binding site). The region of the immunoglobulin that is composed of two heavy chains portions is called the Fc (crystallizable) fragment. The Fc region is responsible for the activation of the immune response, and it is an element that connects the immunoglobulin with the receptors presented on immune system cells (Figure 1) [1,2,3].

Based on differences in the structures of heavy chains, five classes of antibodies have been distinguished: α (IgA), δ (IgD), ε (IgE), γ (IgG) and μ (IgM). The role of all antibodies in the body is to participate in immune reactions. The primary immune response develops at the moment of first contact with the antigen. Then, the body produces primarily IgM antibodies, which are gradually replaced by more specific and more durable IgG antibodies. Interestingly, during the synthesis of all immunoglobulins, normal plasma cells produce a slight excess of kappa (*κ*) and lambda (*λ*) light chains over heavy chains. These small amounts are released into the serum as free kappa or lambda light chains (FLCs) (3.3–19.4 and 5.7–26.6 mg/L, respectively). The production of *κ* chains is approximately double that of *λ* light chains, but because *κ* chains have a monomeric form, their renal clearance is faster than that of dimeric *λ*. As a result of filtration in the glomeruli, FLCs enter the proximal tubules, where they are reabsorbed and metabolized. In physiological conditions, the ratio of *κ* and *λ* free light chains in serum equals 0.26–1.65 [4,5,6].

It has been observed that FLCs are important factors that can trigger inflammation via the activation of mast cells or inhibition of neutrophils apoptosis [7,8]. Therefore, changes in the concentration of FLCs in human body fluids, e.g., blood, urine or cerebrospinal fluid (CSF) can be a sign of various pathologies, and their quantitative determination may be an important element in the diagnosis or prognosis of many diseases [9,10,11,12].

Taking into account the above, the aim of this review was to collect the studies that have been conducted so far concerning free light chains, and to summarize their potential application as a biomarker of various inflammatory diseases.

## 2. Material and Methods

A comprehensive literature search was performed, with the period of time covering studies conducted up to February 2023. The MEDLINE/PubMed database was used to find significant studies. 

The search strategy combined the key words: “free light chains” AND “inflammation”, “monoclonal gammopathy”, “multiple sclerosis”, “viral infections”, “HCV”, “HBV”, “HIV”, “tick-borne disease”, “diabetes”, “cardiovascular diseases/disorders”, “rheumatic diseases”, “rheumatoid arthritis”, “systemic lupus erythematosus”, “Sjogren’s syndrome” or “cancer”.

Studies were limited to studies in English. Finally, 96 publications were included in this review.

## 3. Results 

### 3.1. Monoclonal Gammopathy

Monoclonal gammopathies include not only malignant conditions such as multiple myeloma, but also monoclonal gammopathy of undetermined significance (MGUS), smoldering multiple myeloma (SMM) and AL amyloidosis. So, monoclonal gammopathy is not a single disease entity, but a group of diseases whose common feature is monoclonal immunoglobulin production by an abnormal B-cell clone.

Plasma cells are found in the bone marrow and are formed from B lymphocytes. Their primary function is to produce antibodies—immunoglobulins, which help protect the body against antigens. In a healthy person, plasma cells are produced as needed. They are found in the bone marrow along with maturing red blood cells, platelets and other types of white blood cells. However, sometimes cells start to divide uncontrollably, creating multiple copies. Since these cells come from a single plasma cell, they produce the same antibody, a monoclonal immunoglobulin (a monoclonal protein, or M protein), which is released into bloodstream and may also be excreted in the urine [8,9,13,14,15].

The body of a healthy person produces five classes of immunoglobulin: IgG, IgM, IgA, IgD and IgE. Normal immunoglobulins consist of two identical heavy chains and two identical light chains. Heavy chains determine which class the immunoglobulin belongs to. Unfortunately, in monoclonal gammopathy, monoclonal immunoglobulins consisting of one type of heavy and one type of light chain are generally produced in excess in comparison to other antibodies. In some patients, only light chains are produced [8,9]. FLC assays have been proven to be very important in the initial diagnosis of patients with suspected monoclonal gammopathy, as demonstrated by clinical trials and practice. Moreover, FLC tests also serve as a prognostic indicator for the development of monoclonal gammopathy, i.e., multiple myeloma. Therefore, quantitative measurements of FLCs have been recommended for the diagnosis of patients with plasma cell dyscrasias, i.e., patients with only light chain myeloma with negative results of serum and urine immunofixation, or in patients with very low concentrations of monoclonal protein [15,16].

As was mentioned above, the diagnosis of AL amyloidosis requires evidence of the presence of monoclonal proteins that can be detected by FLC measurements. Amyloidosis is a multi-system ailment that consists of the deposition of abnormal proteins (amyloids) in various tissues and all major organs, e.g., liver, kidneys or heart [17]. Until today, about 30 different proteins that cause amyloidosis have been described. The name of a specific disease subtype usually includes the name of the protein from which amyloid is formed, e.g., light chain amyloidosis [18,19,20,21]. This causes damage to the affected organs, which are very often the kidneys. Moreover, it was suggested that the majority of patients present the *λ* type of monoclonal light chain [22,23]. Literature data have indicated that except for kidneys, the heart is the most frequently affected organ by light chain amyloidosis [24]. In addition, in patients with primary systemic immunoglobulin light chain amyloidosis, the association between cardiac biomarkers, echocardiographic parameters and FLCs was investigated. In patients with markedly elevated levels of *λ*FLCs, the concentrations of the N-terminal prohormone of Brain Natriuretic Peptide and troponin I were increased. Moreover, the correlation between monoclonal *λ*FLCs with all blood markers of heart dysfunction and the value of the diastolic dimension of the interventricular septum suggests that *λ*FLCs may be independent markers of severe damage to cardiomyocytes, as well as poor prognosis [21].

Indisputably however, the clinical significance of FLC determinations in the course of monoclonal gammopathy is well known, and has been confirmed by numerous literature data. Therefore, this review will focus on describing new potential applications of FLCs [4,25,26,27,28,29].

### 3.2. Multiple Sclerosis

Currently, unfortunately, there is no single test for the diagnosis of multiple sclerosis (MS). Therefore, the McDonald criteria, which include imaging tests (e.g., magnetic resonance imaging), clinical symptoms and the assessment of cerebrospinal fluid are used for diagnosis. The analysis of CSF oligoclonal banding (OCB) and interpretation of the results is very often difficult. Diagnostic difficulties result mainly from the fact that multiple sclerosis is an inflammatory disease of the central nervous system with a very diverse course. Therefore, new and early diagnostic indicators of this disease are currently being sought.

For the first time, immunological stimulation leading to enhanced intrathecal synthesis of free light chains was observed in 80 s [30]. FLC synthesis is an early event of MS, so to this day the diagnostic utility of free light chains in multiple sclerosis is being studied mostly due to very promising results and methodological advantages in comparison to OCB analysis. The currently presented results in the literature give real hope for improving the diagnostic standards in MS.

Several studies have shown the importance of FLCs in MS diagnosis. It has been observed that *κ*FLC levels in CSF and serum are many times higher in multiple sclerosis in comparison to patients with other neurological disorders, as well as healthy individuals. Moreover, some studies suggest that the CSF *λ*FLC concentration is also elevated in MS patients, but the levels of lambda light chains are only moderately increased. Due to the fact that *λ*FLCs have a dimeric form and that they are not able to cross the blood-brain barrier (BBB), their elevation may confirm the intrathecal production of immunoglobulins and chronic inflammation [31,32,33,34,35,36,37,38,39].

Some studies have also evaluated the usefulness of FLCs as prognostic markers. It has been suggested that *κ*FLCs alone have no prognostic value [38]. However, it has also been observed that patients with clinically isolated syndrome (CIS) that developed into MS had an increased *κ*FLC index value, which is a combination of *κ*FLCs and albumin in serum and CSF [31,38,39]. Furthermore, as might be expected, *κ*FLC index values were significantly higher in multiple sclerosis in comparison to CIS [31,37,38,39,40,41]. Additionally, Berek et al. showed that in patients with a *κ*FLC index above 100.0, the risk of a second clinical attack within the next one to two years is 2 and 4 times higher, respectively, in comparison to patients with lower values in the index. In addition, Voortman et al. revealed that *κ*FLC index values are lower in patients with nonactive disease in comparison to active. Therefore, it seems that the *κ*FLC index is a promising marker that may predict MS activity [34,40]. Increased value of the *κ*FLC index was also associated with increased risk of relapse [39]. 

Most of the studies which focused on the diagnostic significance of FLCs revealed that the *κ*FLC index has a higher diagnostic sensitivity (93%) than, for example, the IgG index (85%) or OCB (88%). However, the ability of the *κ*FLC index to detect MS seems to be similar to the *κ*FLC concentration in CSF (diagnostic sensitivity: 93% and 96%, respectively) [32]. On the contrary, it has been proven that the *κ*FLC index has lower specificity and sensitivity (95.7% and 93.1%, respectively) than OCBs (98.6% and 96.5, respectively), which may suggest that those tests should be used in a complementary manner [41].

Moreover, what should be pointed out is that the *κ*FLC index is elevated in most patients with the presence of oligoclonal bands [34,37]. The FLC and IgG concentrations in serum and CSF were also combined in *κ*IgG and *λ*IgG indexes. It has been revealed that except for type 2 OCB, the value of the *κ*IgG-index was also increased in type 3 compared to type 1 or 4. It is worth mentioning that in MS, both type 2 and 3 can be seen. The *κ*IgG-index, in the same way as the *κ*FLC index, had high diagnostic accuracy (84.7% and 79.2%, respectively); however, the *κ*IgG-index had a higher ability for MS exclusion with specificity: 80.5% vs. 68.3% [36]. 

Free light chains, especially *κ*FLCs, appear to be promising markers of multiple sclerosis diagnosis. Indisputably, FLCs have very high diagnostic sensitivity and specificity, so they can be used for the detection of MS and differentiation from other neurological disorders. Moreover, in contrast to the analysis of OCBs, FLC determinations are quantitative and allow us to eliminate the risk of interpreting results subjectively. In addition, measurements of FLCs are fast, easy and cost-effective. Therefore, it seems that the quantification of FLCs and calculation of the *κ*FLC index and *κ*IgG-index may enhance the diagnostic standard of MS, all things considered. 

The newest consensus statement concerning the recommendations for MS diagnosis has been published in February 2023. A panel of experts in the management and diagnosis of multiple sclerosis that convened in Vienna, Austria recommend the inclusion of *κ*FLC measurements in the next revisions of multiple sclerosis diagnostic criteria. *κ*FLCs are a promising additional tool that quantitively reflects the intrathecal production of immunoglobulins. Determinations of kappa free light chains in CSF alongside OCB analysis should be complementary tests when the concentration of *κ*FLCs are borderline, or when unequivocal interpretation of the OCB result is difficult or impossible [42].

### 3.3. Viral Infections

Association of free light chains with viral infections, including, for example, hepatitis C virus (HCV), hepatitis B virus (HBV), human immunodeficiency virus (HIV) and Severe Acute Respiratory Syndrome Coronavirus 2 (SARS-CoV-2) have been investigated in various studies.

#### 3.3.1. COVID-19

Nowadays, one of the most investigated infections is SARS-CoV-2. In 2021, Małecka-Giełdowska et al. suggested the application of FLC measurements in distinguishing between severe and non-severe courses of COVID-19. Firstly, scientists observed that FLC levels were markedly elevated in COVID-19 patients in comparison to non-COVID-19 patients, but hospitalized in intensive care units (ICU). Importantly, the *κ*:*λ* ratio was similar in those groups. On the other hand, it was revealed that the *λ*FLC concentration was higher and the *κ*:*λ* ratio was decreased in the SARS-CoV-2-infected but non-hospitalized in ICU group, compared to the non-infected patients from the ICU. There was also a difference in the *κ*FLC concentration and *κ*:*λ* ratio between tested groups with the highest values in COVID-19 patients. Moreover, *κ*FLCs have the highest diagnostic power to distinguish between mild/moderate and severe COVID-19 (sensitivity ~87% and specificity ~93%). This may suggest the intensification of the immune system response in patients with SARS-CoV-2 infection [43]. 

Knowing that SARS-CoV-2 infection induces hyperinflammation and synthesis of immunoglobulins, the correlation between FLCs and specific antibodies against SARS-CoV-2 has been also assessed. The concentration of FLCs was higher in COVID-19 patients in comparison to healthy and non-vaccinated against SARS-CoV-2 patients. In addition, *κ*FLC level was higher in healthy vaccinated patients than in non-vaccinated patients. What is interesting is that FLC concentrations correlated with total IgG, which may be generally explained by the fact that FLCs represent the excess of light chains produced by secreting plasma cells during the increased synthesis of immunoglobulins observed in inflammation. Another interesting observation is that FLC concentrations correlated with specific IgG antibodies to the receptor binding domain (RBD) of the S1 subunit of the spike protein and to the nucleocapsid protein. It means that the FLC level may reflect the acute immune response and subsequent production of specific antibodies after the stimulation by SARS-CoV-2, both after infection and vaccination. Similar to the study by Małecka-Giełdowska et al., the ratio was in normal ranges, but in the vaccinated group the value indicates the increased synthesis of *κ*FLCs [44]. It is presumably caused by the fact that the rearrangement of the genes encoding *λ* light chains is overdue in relation to *κ* [43].

The two studies mentioned above also analyzed the association of IL-6 with free light chains. IL-6 is one of the most important proinflammatory factors associated with viral infections, and became a prognostic and diagnostic marker of COVID-19. The correlation between IL-6 and FLCs, as well as specific anti-SARS-CoV-2 antibodies has been described. This is further proof that the level of FLCs reflect the hyperactivity of the immune system caused by SARS-CoV-2 [43,44].

#### 3.3.2. Hepatitis C

One of the most common complications of viral hepatitis is cryoglobulinemia. The spectrum of HCV-associated cryoglobulinemia varies from asymptomatic to severe vasculitis or lymphoma. Cryoglobulinemia occurs in 10–56% of patients with hepatitis C, while the most common extrahepatic manifestation of hepatitis C is mixed cryoglobulinemia (MC). Its essence is the formation of monoclonal immunoglobulins that react with polyclonal immunoglobulins and activate the complement system.

A recent study on a Brazilian population with HCV infection and MC performed an analysis of free light chains. *κ*FLC levels were increased in the cryoglobulinemia group in comparison to HCV patients without MC. However, the *κ*:*λ* ratio was similar in both tested groups. Interestingly, in this study, the ratio was higher in HCV patients with severe liver fibrosis than in patients without significant pathological changes in the liver. Oliveira et al. also correlated *κ* and *λ*FLCs with immunoglobulin concentrations. There was a relationship between free light chains and IgM, IgG and IgA levels in HCV patients presenting cryoglobulinemia, whereas in the group without cryoglobulinemia, *κ* and *λ* FLCs correlated only with IgG. These results suggest the polyclonal production of immunoglobulins only in HCV individuals presenting cryoglobulinemia. The conclusion is that determination of FLC levels can be used as diagnostic tool for polyclonal B-lymphocyte activation due to chronic HCV infection [45]. 

Apart from HCV-related MC, increased levels of FLCs were also observed in MC-vasculitis and B cell non-Hodgkin lymphoma (B-NHL) [46,47,48]. A correlation between *κ*FLCs, the ratio with the cryoglobulin level and B cell disorder severity has been observed. This suggests that abnormal FLC levels may be a potential prognostic biomarker of MC and B-NHL. In this way, the FLC ratio may be a marker to monitor the treatment response of lymphoproliferation related to HCV [48,49]. For this reason, Basile et al. assessed the FLC levels in HCV patients with MC vasculitis who were treated with rituximab. As alleged, non or partial responders to the rituximab therapy were patients with an abnormal baseline FLC ratio [46]. Moreover, it has been observed that the ratio is increased in almost 50% HCV patients with cryoglobulinemia vasculitis symptom maintenance or recurrence. On the contrary, only 17/100 patients with a complete response to direct-acting antivirals had a ratio above normal ranges (1.65) [50]. 

In a clinical study on asymptomatic and symptomatic cryoglobulinemia patients with HCV, the serological biomarkers were analyzed. The concentrations of *κ* and *λ* FLCs were elevated in both asymptomatic and symptomatic patients [51]. In addition, in one study no significant differences were observed among tested subgroups of patients (without cryoglobulinemia, type II and type III). On the other hand, FLC levels were increased in the total HCV group in comparison to healthy volunteers [52]. Therefore, it was speculated that even in a group of HCV patients without mixed cryoglobulinemia symptoms, the low concentration of cryoglobulins may be responsible for the activation of inflammation, including the activation of B cells [51].

#### 3.3.3. Hepatitis B

According to our best knowledge there was only one study that assessed the FLC concentration in patients with a HBV infection. Taking into account that FLCs may reflect the activation of B cells and inflammation, it was suggested that the level of FLCs may also correlate with the severity of liver damage caused by HBV. It was also suggested that the level of serum FCLs was associated with the disease progression. The level of *κ* and *λ* FLCs increased with the intensity of histological activity, while the ratio was similar. Moreover, FLC levels were the highest in cirrhotic patients in comparison to those with lower stages of fibrosis caused by hepatitis B virus. Interestingly, the *κ*FLC diagnostic accuracy related to the patients with cirrhosis was higher than the currently most studied algorithms: aspartate aminotransferase to platelet ratio index (APRI), or AST to ALT ratio (AAR). Since FLCs are closely associated with an anti-HBV immune response, it was suggested that especially *κ* light chains are promising prognostic markers of HBV [53].

#### 3.3.4. AIDS

Human immunodeficiency virus (HIV) infection is a chronic disease that causes progressive impairment of the immune system of the infected person. HIV belongs to the family of retroviruses and primarily attacks cells of the immune system—white blood cells (CD4 T cells, monocytes, macrophages) located in the blood, bone marrow, digestive tract and central nervous system. Acquired immunodeficiency syndrome (AIDS) usually develops a few years after infection. It is a state of increased susceptibility of the body to all pathogens and an increased risk of developing cancer [54,55].

Currently, there are two main types of the virus: HIV-1 and HIV-2. HIV-1 is responsible for most infections—it occurs in different parts of the world. Increased FLC concentrations, both kappa and lambda, was found in the serum of HIV-1 infected patients. There was also a correlation between FLC levels and the severity or viral load of HIV-1 [56]. Moreover, it has been demonstrated that most patients with HIV-1 infection had an increased production of FLCs in the CSF. Moreover, the prevalence of *λ* dimeric forms of FLCs was observed, which may suggest the local synthesis or disruption of BBB [57,58]. In addition, asymptomatic children and children presenting subacute encephalopathy with coexisting HIV-1 infection had confirmed increased levels of FLCs and interleukin-6 (IL-6) or macrophage colony-stimulating factor (M-CSF) [59]. Therefore, it was suggested that elevation of FLCs, mainly *λ*FLCs, should be taken into account as early markers reflecting pathological processes of the central nervous system.

Measuring levels of FLCs may have several applications, including predicting the risk of AIDS-defining opportunistic infections in HIV patients. Shiels et al. demonstrated that elevated *κ* and *λ* FLCs were associated with AIDS. Furthermore, the polyclonal synthesis of FLCs was shown to be directly correlated with the increased risk of AIDS, whereas monoclonal was not. These findings suggest that measuring free light chains as well as the ratio may be helpful in assessing the predisposition to immune suppression and AIDS development [60]. Moreover, it has been proposed that the changes in FLC concentrations may be useful for the prediction of HIV and AIDS-related lymphomas. An interesting study which was performed by Landren et al. measured the levels of FLCs, IgA, IgM, IgG and monoclonal immunoglobulins in HIV-infected and lymphoma-free (control) patients, as well as patients who developed B-NHL. The samples were taken 0–2 years and 2–5 years before diagnosis of lymphoma. The FLC levels were associated with higher risk of NHL. On the other hand, immunoglobulin level and M proteins were not associated with NHL risk [61]. Similar results were presented by Bibas et al. who measured FLCs before diagnosis of B-cell dysfunctions. The polyclonal production of FLCs was also observed in a group of patients with non-Hodgkin and Hodgkin lymphomas. FLCs, independently of CD4+ count, were useful in predicting the above-mentioned disorders. Moreover, in patients without HIV viremia (>6 months) and decreased concentration of *κ* and *λ* FLCs, a reduced risk of lymphoma development was observed [62]. On the contrary, Title et al. observed that FLC levels did not influence the survival of HIV-infected patients with diagnosed lymphoma. Although FLC concentrations are increased in lymphomas, there is no correlation with FLCs, according to the histological subtypes of lymphomas (Hodgkin’s, diffuse large B-cell, Burkitt) associated with HIV [63].

### 3.4. Tick-Borne Diseases

Tick-borne diseases are one of the most important epidemiological threats to humans and animals. Diseases transmitted by ticks are very dangerous and associated with a high risk of serious complications. The most common diseases caused by ticks are Lyme disease and tick-borne encephalitis (TBE).

#### 3.4.1. Lyme Disease 

Lyme disease is caused by spirochetes of the genus *Borrelia burgdorferi sensu lato*, and in its course, various internal organs can be affected. Non-specific general symptoms and diverse clinical presentation, as well as problems with laboratory diagnostics, often cause difficulties in the diagnosis of Lyme disease. The measurements of free light chains in the CSF were proposed as novel markers of Lyme neuroborreliosis. The *κ*FLC index and *λ*FLC index have been calculated as a combination of FLCs with the level of albumin (serum and CSF). The FLC quotient has been also calculated as a ratio of serum and CSF FLCs. To calculate the total IgM index, the values of IgM and albumin in serum and CSF were needed. Significant differences for tested parameters were observed between patients with neuroborreliosis and control groups (patients with inflammatory and non-inflammatory neurological disorders). The sensitivity of the intrathecal fractions of *κ* and *λ* FLCs reached up to 87% in Lyme neuroborreliosis patients. Additionally, the diagnostic sensitivity of the *κ* and *λ* FLCs indexes was very high (88.0 and 100.0%, respectively). However, the elevation of FLCs in neuroborreliosis as well as in other inflammatory neurological disorders may cause difficulties in interpretation of the results [64,65].

#### 3.4.2. Tick-Borne Encephalitis

In turn, tick-borne encephalitis virus (TBEV), which is transmitted by ticks, may cause tick-borne encephalitis. TBE is an infectious disease that affects the nervous system, and has a biphasic course. The 1st phase usually lasts up to nine days and resembles a cold. The 2nd phase of TBE lasts from several weeks up to months and is accompanied by, for example, high fever, severe headaches and dizziness, vomiting or paresis. Disturbances or loss of consciousness and meningeal symptoms may also occur. The inflammation caused by TBEV is associated with increased production of immunoglobulins, including specific antibodies against the virus. Therefore, FLC levels were evaluated in patients with TBE, both before and after treatment. *λ*FLCs were presented in increased amounts in pre-treatment serum samples. On the other hand, except for specific IgM and IgG antibodies, *λ*FLCs in the CSF as well as the *κ*FLC index and *λ*FLC index were elevated after treatment, which is worth emphasizing. Moreover, free light chain concentrations and indexes correlated with serum IgG TBEV antibodies and CSF IgM TBEV antibodies. Additionally, serum *κ*FLCs correlated with *λ*FLCs in the CSF. The increased levels of FLCs may reflect increased immunoglobulin synthesis. On the other hand, elevated amounts of FLCs in the CSF and decreased serum *λ*FLC concentrations after treatment may indicate blood-brain barrier (BBB) dysfunction or damage, as well as intrathecal synthesis of specific antibodies against TBEV [65].

### 3.5. Diabetes

Diabetes is a civilization metabolic disease characterized by increased blood glucose levels. Diabetes is associated with abnormal secretion and/or action of insulin in the body. In type 1 diabetes (insulin-dependent), the chronic autoimmune process leads to the gradual destruction of the insulin-producing β cells of the pancreas. On the other hand, in type 2 diabetes (T2D) chronic inflammation as well as changes in the level of proinflammatory factors and activation of various leukocyte populations are observed [66]. 

Since free light chains could be biomarkers of immune responses and inflammation [67], FLC measurements in type 2 diabetes have been performed. It has been revealed that both *κ*FLCs and the ratio were significantly lower in T2D in comparison to the healthy population, whereas the *λ*FLC concentration was increased in diabetic patients. Interestingly, the area under the receiver operating characteristic curve (AUC ROC) was the highest for the ratio (0.996), and what should be pointed out is that it was higher in comparison to glycated hemoglobin A1c (HbA1c). Moreover, the *κ*:*λ* ratio at a cut-off point of 1.3 seems to be very good biomarker to differentiate healthy from diabetic patients (96% sensitivity and negative predictive value (NPV), and 100% specificity and positive predictive value (PPV)) [68].

In addition, it has been observed that the sum of *κ* and *λ* FLCs (combined: cFLCs) is associated with the degree of atherosclerotic transformation of the carotid artery in type 2 diabetes [69]. The risk of cardiovascular disease (CVD) events in patients with diabetes is higher in those showing high concentrations of FLCs, simultaneously with systolic blood pressure and triglyceride levels. Moreover, cFLCs were suggested to be a more accurate predictor of CVD events in comparison to high-sensitivity C-reactive protein (hsCRP) [70,71].

Diabetes causes also changes in glomeruli that cause increased permeability of glomerular blood vessels. Diabetic kidney disease (DKD) develops as a result of increased blood glucose levels. As a novel marker of early DKD, FLCs have been taken into account. A high level of polyclonal FLCs was seen in the serum of diabetic patients before the development of kidney impairment. Interestingly, South Asian diabetic patients had higher concentrations of FLCs than Caucasian patients. It may be associated with a higher rate of subclinical signs of inflammation in South Asian diabetics and genetic differences in both groups. The elevated *κ* and *λ* FLCs were related to the concentration of cystatin C and other markers reflecting renal functions. The correlation between increased serum FLCs and decreased eGFR was also reported. Moreover, an increased FLC level in urine was observed, and it seems to be associated with the albumin to creatinine ratio (ACR). What should be pointed out is that patients with normal ACR and albumin concentrations had abnormal ratios of FLCs:creatinine and *κ*FLCs, respectively. In this manner, FLCs may be used for the early prediction and diagnosis of DKD [72]. 

### 3.6. Cardiovascular Disorders

Cardiovascular diseases are currently one of the most common causes of death in the world. Many of them develop for a long time with a barely noticeable symptoms. When the first symptoms become visible, it is often too late to implement effective treatment. Therefore, it is very important to find an early biomarker of cardiovascular diseases. 

Serum free light chains have been assessed as potential biomarkers for the diagnosis of CVD. Analysis of FLCs and hsCRP has been performed in patients with atherosclerosis, ischemic heart disease (IHD) and healthy controls. A high level of FLCs was demonstrated in patients with IHD; however, there was no correlation with hsCRP [73]. Another study on 628 patients demonstrated that the concentrations of FLCs and other cardiac biomarkers such as B-Type Natriuretic Peptide (BNP), hsCRP and lymphocyte count were independent predictors of mortality in patients with decompensated heart failure [74]. In addition, there was another study that described the association between FLC levels and ST elevation myocardial infraction (STEMI). The results of that study revealed that in patients with STEMI, FLC concentrations correlate with left ventricle ejection fraction. In addition, more specifically reduced systolic function was observed in patients with increased levels of combined FLCs [75]. A study by Shantsilla et al. focused on patients with acute heart failure (HF), stable heart failure and stable coronary artery disease, but without HF. The elevated level of cFLC in acute HF correlated with creatinine and cystatin C concentrations. The results also demonstrated that cFLC concentration, in contrast to BNP, did not change during the 3-month follow up period. Additionally, further Cox regression analysis showed that increased cFLC concentration is associated with the risk of readmission to the hospital or death [76]. 

### 3.7. Rheumatic Diseases

The list of rheumatic diseases is long, but the most common are rheumatoid arthritis (RA), systemic lupus erythematosus (SLE) or Sjogren’s syndrome (SS). Rheumatic diseases are a group of inflammatory disorders where chronic inflammation of the connective tissue is very typical. They have a hidden autoinflammatory base, which means that they are caused by an excessive, incorrect reaction of the immune system. In autoimmune diseases, an overreactive immune system produces antibodies that attack their own tissues [77]. Therefore, free light chains have been evaluated in patients with rheumatoid disorders. 

FLCs have been found to be elevated in RA patients. The concentrations of FLCs were significantly increased in patients with active RA and before symptoms appear, in comparison to RA in remission or healthy controls [78,79,80]. The dysfunction and increased activity of B cells lead to the increased production of immunoglobulins, and as a result, FLCs in patients with RA. In addition, FLC concentrations correlated with the Disease Activity Score 28 and FLCs [81]. On the contrary, the concentrations of FLCs were lower in primary Sjogren’s syndrome in comparison to rheumatoid arthritis, but higher than in healthy controls. Moreover, among the patients with increased FLCs, more than 50% had increased synthesis of *κ*FLCs. In contrast to total IgG or total gammaglobulins, FLCs correlated with extraglandular involvement. Therefore, it can be used as a marker of systemic complications in the primary SS patient group [78,80,82]. On the other hand, it has been suggested that FLC determination had no diagnostic value in comparison to anti–Sjögren’s-syndrome-related antigen A (anti-SSA) antibodies. Both in RA and SS, concentrations of FLCs decreased after treatment [66]. FLC levels were also measured in saliva and serum of SS patients with neurological symptoms (Neuro-Sjögren) and neurological patients without the anti-SSA (Ro) antibodies (controls), but there were no differences in FLC concentrations between SS patients and control group [83]. 

Patients with SLE also have increased concentrations of free light chains that fall below normal ranges after treatment. Moreover, in contrast to *λ*FLCs, among SLE flare patients the concentrations of complement system proteins C3 and C4 are decreased [84,85]. Interestingly, FLCs correlate with CRP, Systemic Lupus Erythematosus Diseases Activity Index (SLEDAI) and Visual analogue scale VAS scores [80,84,85,86,87]. On the contrary, other inflammatory markers such as IL-10 or INF-α were not associated with disease activity [88]. 

### 3.8. Cancers

Local inflammation which can spread to the entire body is associated with the presence of factors that trigger the immune system reaction. In addition to infections or ischemia leading to tissue damage, tumors may also cause uncontrolled inflammation. The human body’s response to the presence of this pathological change is acute phase reaction. Currently, it is well known that the presence of tumors is associated with immune system reaction [89]. Therefore, free light chains have been also studied in patients with tumors. Currently, the level of FLCs has been measured in few cancers, apart from multiple myeloma. However, examples of cancers that are associated with FLC concentration also include lung, breast or gastric cancer. 

The importance of FLCs in cancer development is still poorly understood. However, it has been observed that FLCs can trigger the activation of mast cells. On the other hand, mast cells may have pro-tumorigenic effects, including participation in the stimulation of angiogenesis, degradation of the extracellular matrix or immunosuppression reactions through the secretion of inflammatory mediators. Therefore, it seems that cancer development may be indirectly related to FLCs by affecting mastocytes [90,91]. 

The concentrations of FLCs have been evaluated in serum and bronchoalveolar fluid, and it has been noted that in patients with lung disorders, including non-small cell lung cancer, the concentrations of FLCs are increased. Moreover, overexpression of FLCs was observed in the areas of mastocyte infiltration and was related to poor clinical outcome [90]. 

The increased expression of FLCs was also observed in breast cancer. Interestingly, *λ*FLCs were predominantly localized in stromal inflammatory cells, whereas *κ*FLCs were mostly presented in the cytoplasm of breast cancer cells. The age or tumor recurrence was not associated with FLC expression. On the other hand, there was a correlation between FLCs and tumor size and grade or clinical stage. Importantly, *λ*FLC expression was also associated with poor prognosis of patients with breast cancer. Importantly, it was suggested by Kormelink et al. that inhibition of FLC-mediated mast cell pro-tumorigenic activation may lead to the reduction of tumor growth [91]. 

The increased level of *λ*FLCs was also observed in patients with bone relapse of breast cancer. In addition, decreased RNA levels of *κ*FLCs have been suggested as a predictor of metastasis-free survival and good response to the neoadjuvant therapy of breast cancer [92]. In another paper, an interesting case of a woman with bone pain that had undergone an operation for breast cancer a few years before has been described. The patient’s symptoms suggested a bone relapse of breast cancer, but the treatment was ineffective. Therefore, some laboratory tests were performed. The *κ*:*λ* ratio in this patient was below the reference range, suggesting the monoclonal synthesis of lambda light chains. The serum IF revealed the presence of *κ*IgG and *λ*FLCs in the beta zone. In parallel, the presence of monoclonal *λ* free light chains was confirmed by urine immunofixation (IF). On the other hand, a slight decrease in *λ*FLCs after treatment was observed. The presented case is evidence that IF is a very sensitive method for detecting free light chains, and that FLC determinations may be useful in the differentiation of diseases, e.g., bone relapse with light chain multiple myeloma [93]. 

As is well known, mast cells are also involved in the process of bowel disease development. Consequently, FLCs play an important role in mast cell-dependent colitis [94]. Studies on murine models with inflammatory bowel diseases and colitis revealed that FLC levels in serum and tissues are markedly elevated [94,95]. Moreover, it is well known that colitis, described as chronic inflammation, increases the risk of colitis-associated carcinoma (CAC); it has been observed that in CAC, levels of FLCs are higher in comparison to the control group. Knowing that FLCs may activate mast cells and extend the survival of neutrophils, Ma et al. tried to inhibit FLCs. Using Peptide F991 they observed that tumor formation was inhibited. Moreover, blocking FLCs resulted in the prolongation of survival time in the CAC group. These findings revealed that FLCs may be involved in the transformation of inflammation to cancer [80]. In addition, the rearrangement of the unique V*κ*4-1/J*κ*3 pattern with *κ* light chains is overexpressed in colon cancer cells. Moreover, V*κ*4-1/J*κ*3-FLC may deposit in an insoluble form in the extracellular matrix (ECM). In vivo and in vitro studies have shown that the above-mentioned pattern promotes migration, metastasis and proliferation of cancer cells, as well as activation of the FAK signaling pathway by interaction with integrin β1 [96]. 

The significance of FLCs as biomarkers of inflammatory diseases has been summarized in Table 1.

## 4. Conclusions

In summary, inflammation plays a key role in the development of various diseases, and abnormal levels of free light chains were observed in several inflammatory diseases. The above-described findings underline the importance of free light chains in the diagnosis and prognosis of various inflammatory diseases.

First of all, and most importantly, it seems that the discussed free light chains are very promising complementary tools in addition to the analysis of CSF oligoclonal banding in patients with multiple sclerosis. Secondly, viral infections could lead to increased production of immunoglobulin FLCs. On the other hand, increased levels of FLCs in viral infections may reflect the production of specific antibodies against infections such as SARS-CoV-2. In addition, abnormal concentrations of FLCs were independently associated with the risk of cardiovascular disorder mortality or development of diabetic kidney disease in patients with type 2 diabetes. FLCs have also been found to be increased in rheumatic diseases and related to disease activity. What is very important is that it has been suggested that inhibition of FLCs would reduce the progression of tumorigenesis. Therefore, free light chains may be also a potential therapeutic option for the treatment of selected inflammatory disorders. Indisputably however, further research is needed to confirm the potential clinical significance of free light chains.

## Figures and Tables

**Table 1:** The significance of FLCs as biomarkers of inflammatory diseases.

Disease	Level of FLCs	Application	References
Monoclonal gammopathies	↑ in serum, urine abnormal ratio	▪ diagnosis ▪ prognosis ▪ monitoring response to therapy	[4,25,26,27,28,29]
Multiple sclerosis	↑ in serum, CSF ↑ values of *κ*FLC index and *λ*FLC index	▪ diagnosis (*κ*FLC index has higher sensitivity than IgG-index, but similar to OCBs) ▪ prognosis ▪ prediction of e.g., future attacks or relapses ▪ detection of intrathecal synthesis of immunoglobulins ▪ differentiation from other neurological disorders	[31,32,33,34,35,36,37,38,39]
SARS-CoV-2 infection	↑ in serum abnormal ratio	▪ diagnosis ▪ differentiation between mild/moderate and severe COVID-19 course ▪ differentiation between healthy vaccinated and non-vaccinated patients ▪ reflection of specific anti-SARS-CoV-2 antibodies synthesis	[43,44]
HCV	↑ in serum abnormal ratio	▪ diagnosis of mixed cryoglobulinemia or non-Hodgkin lymphoma ▪ prediction of cryoglobulinemia or liver fibrosis ▪ monitoring response to therapy	[45,46,47,48,49,50,51,52]
HBV	↑ in serum	▪ prediction of cirrhosis ▪ correlation with the stage of liver fibrosis ▪ prognosis ▪ potential treatment application	[53]
HIV	↑ in serum, CSF	▪ prediction of pathological processes of the CNS ▪ prediction of AIDS development or lymphomas ▪ reflection of HIV-1 viral load	[56,57,58,59,60,61,62,63]
Lyme disease	↑ in serum, CSF ↑ values of *κ*FLC index and *λ*FLC index	▪ diagnosis (*κ*FLC index and *λ*FLC index had higher sensitivity than OCBs and IgG-index) ▪ differentiation between neuroborreliosis and other neurological disorders	[64]
Tick-borne encephalitis	↑ *λ*FLCs in serum (pre-treatment) ↑ *λ*FLCs in CSF (post-treatment)	▪ prediction of BBB dysfunction ▪ detection of intrathecal synthesis of immunoglobulins ▪ reflection of specific anti-TBEV antibodies synthesis ▪ monitoring response to therapy	[65]
Diabetes	↓ *κ*FLCs in serum and ratio in T2B ↑ in serum	▪ diagnosis ▪ prediction of CVD events or DKD development	[68,69,70,71,72]
Cardiovascular disorders	↑ in serum	▪ diagnosis ▪ marker of cardiomyocytes damage ▪ predictor of mortality ▪ correlation with ventricle ejection fraction ▪ more accurate predictor of CVD events in comparison to hsCRP	[19,20,21,73,74,75,76]
Rheumatoid arthritis	↑ in serum	▪ prediction of mortality ▪ association with disease activity ▪ differentiation between active and inactive RA ▪ monitoring response to therapy	[78,79,80,81]
Sjogren’s syndrome	↑ in serum abnormal ratio	▪ correlation with extraglandular involvement ▪ marker of systemic complications ▪ monitoring response to therapy	[82,83,86]
Systemic lupus erythematosus	↑ *λ*FLCs	▪ association with disease activity ▪ marker of SLE flare ▪ differentiation between active and inactive SLE	[63,84,87]
Lung cancer	↑ in serum and bronchoalveolar fluid ↑ expression	▪ clinical outcome	[90]
Breast cancer		▪ correlation with tumor size, grade and clinical stage ▪ prediction of poor prognosis and metastasis free-survival ▪ association with bone relapse of breast cancer ▪ monitoring response to therapy ▪ potentially useful in treatment	[91,93]
Bowel diseases	↑ in serum ↑ expression	▪ prediction of colitis-associated carcinogenesis ▪ potentially useful in treatment	[95,96]

FLCs, free light chains; ↑, increased; ↓, decreased; CSF, cerebrospinal fluid; HCV; hepatitis C virus; HBV, hepatitis B virus; HIV, human immunodeficiency virus; TBEV, tick-borne encephalitis virus; CVD, cardiovascular disease; DKD, diabetic kidney disease; RA, rheumatoid arthritis; SLE, systemic lupus erythematosus.
